# Quercetin effectively improves LPS-induced intestinal inflammation, pyroptosis, and disruption of the barrier function through the TLR4/NF-κB/NLRP3 signaling pathway *in vivo* and *in vitro*

**DOI:** 10.29219/fnr.v66.8948

**Published:** 2022-12-30

**Authors:** Hui-Xin Zhang, Ye-Ye Li, Zhong-Jie Liu, Jiu-Feng Wang

**Affiliations:** Department of Veterinary Clinic Medicine, College of Veterinary Medicine, China Agricultural University, Beijing, P. R. China

**Keywords:** quercetin, LPS, pyroptosis, TLR4, NLRP3, GSDMD, interleukins, ZO-1, claudins, IBD

## Abstract

**Background:**

Inflammatory bowel diseases are characterized by the alterations of the mucosa and gastrointestinal physiology, and the core of these alterations is endothelial cells. Quercetin is a flavonoid presents in some traditional Chinese medicine, plants, and fruits. Its protective effects in several gastrointestinal tumors have been demonstrated, but its effects on bacterial enteritis and pyroptosis-related diseases have rarely been studied.

**Objective:**

This study aimed to evaluate the effect of quercetin on bacterial enteritis and pyroptosis.

**Design:**

*In vitro* experiments were performed using rat intestinal microvascular endothelial cells divided into seven groups: control group (no treatment), model group (10 μg/mL lipopolysaccharide (LPS)+1 mM adenosine triphosphate [ATP]), LPS group (10 μg/mL LPS), ATP group (1 mM ATP), and treatment groups (10 μg/mL LPS+1 mM ATP and 5, 10, and 20 μM quercetin). The expression of pyroptosis-associated proteins, inflammatory factors, tight junction proteins, and the percentage of late apoptotic and necrotic cells were measured. *In vivo* analysis was performed using specific pathogen-free Kunming mice pretreated with quercetin and the water extract of *Cacumen Platycladi* for 2 weeks followed by 6 mg/kg LPS on day 15. Inflammation in the blood and intestinal pathological changes were evaluated.

**Results:**

Quercetin used *in vitro* significantly reduced the expression of Toll-like receptor 4 (TLR4), NOD-like receptor 3 (NLRP3), caspase-1, gasdermin D, interleukin (IL)-1β, IL-18, IL-6, and tumor necrosis factor-α. It also inhibited phosphorylation of nuclear factor-kappa B (NF-κB) p65 and increased cell migration and the expression of zonula occludens 1 and claudins, while reduced the number of late apoptotic cells. The *in vivo* results showed that *Cacumen Platycladi* and quercetin significantly reduced inflammation, protected the structure of the colon and cecum, and prevent fecal occult blood induced by LPS.

**Conclusions:**

These findings suggested the ability of quercetin to reduce inflammation induced by LPS and pyroptosis through TLR4/NF-κB/NLRP3 pathway.

## Popular scientific summary

- The Chinese herbal medicine *Cacumen Platycladi* rich in quercetin as well as quercetin used *in vivo* in a mouse model significantly reduces inflammation, protects the structure of the colon and cecum, and prevents fecal occult blood induced by LPS.
- Quercetin used *in vitro* protects the integrity of the tight junction barrier and exerts a regulatory effect on the NLRP3 inflammasome.
- The Chinese herbal medicine rich in quercetin is effective against bacterial enteritis and pyroptosis-related diseases.

Intestinal mucosal capillaries are involved in the integrity of the intestinal barrier and regulate the circulation of metabolic waste in the blood by nourishing the intestinal mucosa (1). Their damage increases vascular permeability and induces endothelial damage (2). The microvascular alteration precedes epithelial disorders in some colitis conditions (3). Bacteria are the main cause of infectious enteritis (4), with diarrhea as the most common sign, while severe cases of endotoxemia or sepsis may cause death. Exogenous bacteria cause the recruitment of many inflammatory cells and mediators after entering the intestinal blood circulatory system, resulting in a series of inflammatory reactions and tissue damage (5).

Pyroptosis is a programmed cell death different from apoptosis, and both are different from necrosis. Indeed, pyroptosis is an inflammatory form of lytic programmed cell death occurring after the infection with intracellular pathogens (6). Both pyroptosis and apoptosis undergo chromatin condensation, but apoptosis is characterized by the rupture of the nucleus into multiple chromatin bodies, while the nucleus remains intact in pyroptosis (7). In addition, pores are formed on the plasma membrane during pyroptosis, causing water influx and cell lysis. Pyroptosis is linked to the development of bacterial enteritis (8). The expression of NOD-like receptor 3 (NLRP3) inflammasome is a typical feature of infectious diseases and a critical protein involved in pyroptosis (9). Its activation requires two signals, such as a pathogen and adenosine triphosphate (ATP). Pathogenic signals, such as Gram-negative bacterial lipopolysaccharide (LPS), are recognized by Toll-like receptor 4 (TLR4), which activates nuclear factor-kappa B (NF-κB) and the downstream NLRP3 inflammasome, thereby causing inflammation and the production of the pro-forms of interleukin (IL)-1β and IL-18 (10, 11). The second signal ATP (12, 13) changes the ion concentration or pH and other factors, activating caspase-1 that cleaves IL-1β, IL-18, and gasdermin D (GSDMD). This process induces the recruitment of more inflammatory factors, thereby enhancing the inflammatory response, which causes the pyroptosis of microvascular endothelial cells (14). The apoptosis-associated (ASC) speck-like protein is localized mainly in the nucleus of monocytes and macrophages, but it moves rapidly to the cytoplasm, perinuclear space, endoplasmic reticulum, and mitochondria in the presence of pathogen infection, and it is a key adaptor protein in the activation of the inflammasome.

Zonula occludens 1 (ZO-1) is an important tight junction protein. Its downregulation or decreased activity affects the formation of tight junction between cells (15), consequently affecting the normal function of the intestinal barrier. It also increases the risk of bacterial enteritis, which is caused by harmful bacteria penetrating the intestinal mucosa and entering the bloodstream (16). The bacterial toxins, inflammatory factors, and tumor necrosis factors (TNFs) directly damage the tight junction and affect the activity and distribution of ZO-1 (17, 18). In addition, claudin-1 and claudin-2 are tight junction proteins expressed in both endothelial and epithelial cells and involved in the development of tissue barriers between different tissue compartments by the regulation of the efflux of molecules through tight junction complexes (19).

Quercetin is an effective ingredient of plants widely used in the traditional Chinese medicine, including *Cacumen Platycladi*, and it is naturally present in the flowers, leaves, and fruits of many plants. It has inhibitory effects on the proliferation, metastasis, apoptosis, and metabolism of tumor cells through multiple signaling pathways, in numerous cancer types (20–24). Quercetin promotes and protects the function of the intestinal tight junction barrier (25) and degrades some intestinal bacteria, decreasing their amount in a time-dependent manner (26). It also protects human retinal microvascular endothelial cells from the high-glucose-induced injury by inhibiting the expression of NLRP3, caspase-1, IL-1 β, and IL-18 (27) and inhibits the expression of NLRP3, caspase-1, iNOS, and inflammatory factors in the LPS-activated mononuclear macrophages, consequently improving their antioxidant and anti-inflammatory abilities (28). Therefore, quercetin protects the integrity of the tight junction barrier and exerts a regulatory effect on the NLRP3 inflammasome.

## Materials and methods

### Drugs and reagents

Quercetin (Batch No. Z100081) was purchased from the National Institute for Food and Drug Control (Beijing, China). The leaves of *Cacumen Platycladi* were purchased from Nanjing Tongrentang (Nanjing, China). Dulbecco’s Modified Eagle Medium (DMEM), fetal bovine serum (FBS), and trypsin were purchased from BasalMedia (Shanghai, China). LPS and ATP were purchased from Sigma-Aldrich.

### Cell culture

Rat intestinal microvascular endothelial cells (RIMVECs) were obtained from the isolation of primary cells from rat primary intestinal mucosal tissues and subsequently subjected to stable passaging. The cells were cultured in a complete standard medium for RIMVECs, such as high glucose DMEM supplemented with 10% FBS (Gibco, Grand Island, NY, USA) and 1% penicillin-streptomycin mixed solution (Solarbio, Beijing, China). The cells were routinely incubated at 37°C in a humidified incubator under 5% CO_2_ (29).

### Effect of quercetin on cell viability

RIMVECs were seeded in a 96-well cell culture plate at a concentration of 1 × 10^5^ cells/well in 100 μL DMEM per well. When the cells reached 80% confluency, they were treated with quercetin at different concentrations (300, 160, 80, 40, 20, 10, 5, 2.5, and 1.25 μM) and incubated for 48 h. DMEM was discarded at the end of the incubation time, Cell Counting Kit-8 solution (Beyotime Biotechnology, China) in fresh DMEM was added, and the cells were incubated at 37°C for 40 min in the dark. The absorbance was measured at 450 nm using a microplate reader (Thermo Scientific, Waltham, MA).

### *In vitro* experiment

Cells were seeded in a 96-well cell culture plate at a concentration of 1 × 10^5^ cells/well in 100 μL DMEM per well. When the cells reached 80% confluency, they were divided into different groups: control group (no treatment), model group (10 μg/mL LPS 24 h followed by 1 mM ATP 4 h), LPS group (10 μg/mL LPS 24 h), ATP group (1 mM ATP 4 h), and treatment groups (10 μg/mL LPS simultaneous to quercetin 5, 10, and 20 μM 24 h followed by 1 mM ATP 4 h). Cell viability was measured after 24 h using the Cell Counting Kit-8 (CCK-8) kit according to the previous paragraph.

### Western blot

RIMVECs were seeded, divided into groups, and treated as described in the *in vitro* experiment paragraph. Total proteins were extracted using the radio immunoprecipitation assay (RIPA) cell lysis buffer for 10 min, and protein concentration was determined using the bicinchoninic acid (BCA) protein detection kit (Beyotime Biotechnology, China). A standard western blot protocol was performed, and the following primary antibodies were used overnight at 4°C: TLR4 (1:800) (ABclonal, Wuhan, China), NLRP3 (1:1000) (ABclonal), GSDMD (1:1000) (ABclonal), caspase-1 (1:1000) (ABclonal), ASC (1:1000) (ABclonal), NF-κB p65 (1:1000) (Abmart, Shanghai, China), p-NF-κB p65 (1:400) (Abmart), p-I-κB (1:1000) (Abmart), I-κB (1:1000) (Abmart), IL-18 (1:1000) (Affinity Biosciences, Colorado, USA), IL-1β (1:1000) (Affinity Biosciences), claudin-1 (1:1000) (Affinity Biosciences), claudin-2 (1:1000) (Affinity Biosciences), ZO-1 (1:1000) (Affinity Biosciences), and β-tubulin (1:1000) (Affinity Biosciences) used as loading control, followed by the incubation with HRP (Horseradish Peroxidase)-conjugated secondary antibodies (1:5000) (ABclonal) for 1 h at room temperature. The blots were visualized using a Tanon 5200 chemiluminescence imaging system (Shanghai, China) and quantified using the ImageJ software (National Institutes of Mental Health, Bethesda, MD, USA).

### Enzyme-linked immunosorbent assay

RIMVECs were seeded in 12-well cell culture plate at a concentration of 1 × 10^5^ cells/well in 1.5 mL DMEM. When the cells reached 90% confluency, they were divided into the same groups as described in the *in vitro* experiment paragraph. The supernatant was collected after 24 h, and IL-1β, IL-18, IL-6, and TNF-α protein contents in the supernatant were measured according to the manufacturer’s instructions of their respective enzyme-linked immunosorbent assay (ELISA) kits (Jiangsu Enzyme Immunoassay Industry Co., Ltd., Jiangsu, China).

### RNA extraction and quantitative reverse transcription PCR

RIMVECs were seeded in a 6-well cell culture plate at a concentration of 1 × 10^5^ cells/well in 2 mL DMEM per well. When the cells reached 90% confluency, they were divided into control group, model group (LPS+ATP), and quercetin groups at the doses described in the paragraph of the *in vitro* experiment and incubated for 12 h. Total RNA Extraction kit and reverse transcriptase were purchased from Aidlab (Beijing, China). Reverse transcription was performed using the Prime Script RT reagent kit (TAKARA). Quantitative reverse transcription PCR (RT-qPCR) was performed using a CFX9600 apparatus (Bio-Rad, Hercules, CA, USA). The RT-qPCR conditions were as follows: pre-denaturation at 94°C for 30 s; 40 cycles of denaturation at 94°C for 5 s, annealing at 60°C for 15 s. The 20 μL reaction mixture was composed of 0.4 μL (10 μmol/L) forward primer, 0.4 μL (10 μmol/L) reverse primers, 0.8 μL cDNA, 8.4 μL dd.H_2_O, and 10 μL qPCR SuperMix. The gene sequences of rat-derived *TLR4*, *GSDMD*, *caspase-1*, *IL-1β*, and *IL-18* genes were confirmed in GenBank. Primer Premier 5.0 software was used to design the primers, which were then synthesized by Sangon (Shanghai, China). The target gene expression was calculated by normalizing it with the expression of Glyceraldehyde-3-phosphate dehydrogenase *(GAPDH)* using the 2^−ΔΔCt^ method. The specific primer sequences of the target genes are listed in Table 1.

**Table 1:** Primer sequences.

Target gene	Primer sequence (5' to 3')	Length (bp)
IL-18-1F	ACCGCAGTAATACGGAGCAT	20
IL-18-1R	TCTGGGATTCGTTGGCTGTT	20
Caspase-1-1F	TGGAGCTTCAGTCAGGTCCAT	21
Caspase-1-1R	ATGCGCCACCTTCTTTG TTC	20
TLR4-1F	ATGCCTCTCTTGCATCTGGC	20
TLR4-1R	TAGGAAGTACCTCTATGCAGGGAT	24
IL1-6F	CAGCTTTCGACAGTGAGGAGAA	22
IL1-6R	TCTTGTCGAGATGCTGCTGT	20
GSDMD-2F	AGATCGTGGATCATGCCGTC	20
GSDMD-2R	AGGGCTTGAAGCTGGTAGAAT	21

### Confocal fluorescence microscopy

The sterilized coverslips were placed into the wells of a 24-well plate, and then RIMVECs were added to each plate at the concentration of 1 × 10^5^ cells/well in 600 μL DMEM. When the cells reached 90% confluence onto the coverslip, they were divided into the same groups as described in the *in vitro* experiment paragraph. At the end of the incubation time, the cells were fixed with 500 μL paraformaldehyde (PFA) for 40 min, followed by permeabilization with 0.2–0.5% Triton X-100 for 20 min. The cells were washed three times with phosphate buffered saline (PBS) before and after each step and then incubated with the following primary antibodies at 4°C overnight: NLRP3 (1:1000, ABclonal), GSDMD (1:1000, ABclonal), caspase-1 (1:1000, ABclonal), and ZO-1 (1:1000, Affinity Biosciences). Next, the secondary antibody (1:5000 dilution in 5% BSA, ABclonal) was added, and the cells were incubated at room temperature for 1 h in the dark. One μg/mL 4’,6-diamidino-2-phenylindole (DAPI) was added to each well, and the cells were incubated at room temperature for 20 min in the dark. After washing, the coverslips were removed from the wells and blotted to remove any excess water. Each coverslip was placed onto a microscope slide, and the slides were sealed with nail polish and observed under a confocal fluorescence microscope (NIKON Ti-S, Tokyo, Japan).

### Flow cytometry

The sterilized coverslips were placed into the wells of a 6-well plate, and then RIMVECs were added to each well at the concentration of 1 × 10^5^ cells/well in 2 mL DMEM. Cells were then divided into control group, model group (LPS+ATP), and quercetin groups at the doses and incubation times described in the paragraph of the *in vitro* experiment. The adherent cells were digested with trypsin, centrifuged at 300 g for 5 min, and the supernatant was removed. The cells were mixed with 0.1% BSA in a 1 × 10^7^ cells/mL suspension. The Fc receptor blockers were used to reduce the non-specific staining. Then, the cells were treated with Annexin V/3,8-Diamino-5- [3-(diethylmethylammonio) propyl]-6-phenylphenanthridinium diiodide (PI) and incubated for 30 min at 4°C in the dark as described in the manufacturer’s instructions. The cells were resuspended in cell staining buffer for the analysis with flow cytometry.

### Cell scratch assay

Several horizontal lines were drawn with a marker pen on the backside of a 6-well plate (a line was drawn every 0.5–1 cm across the well and at least five lines for each well). The RIMVECs at a concentration of 5 × 10^5^ cells/mL were added to each well in 2 mL DMEM, and 100% cell confluence was reached after the overnight incubation. The next day, the layer of confluent cells was scratched using a pipette tip according to the marked line. After washing three times with PBS, the cells were cultured in a fresh serum-free DMEM, divided into control group, model group (LPS+ATP), and quercetin groups at the doses and incubation times described in the paragraph of the *in vitro* experiment and incubated at 37°C under 5% CO_2_. The cells were observed under a microscope and photographed using TS-View. The ImageJ software was used to process the pictures (6–8 horizontal lines were randomly drawn between the scratches for calculating the average distance between cells).

### Inhibition of NLRP3 by MCC950

RIMVECs were seeded in a 96-well cell culture plate at a concentration of 1 × 10^5^ cells/well in 100 μL DMEM per well. When the cells reached 80% confluency, they were treated with MCC950 at different concentrations (0, 0.05, 0.5, 5, 10, and 50 μM), according to a previous report (30) and incubated for 12 h. Cell viability was measured using Cell Counting Kit-8 solution (Beyotime Biotechnology), as explained in the paragraph of cell viability. As regards the viability under different treatments, cells were seeded in a 6-well plate at a concentration of 1 × 10^5^ cells/well in 200 μL DMEM per well. When the cells reached 80% confluency, they were divided into the following groups: control group (no treatment), model group (10 μg/mL LPS 24 h followed by 1 mM ATP 4 h), treatment groups (10 μg/mL LPS simultaneous to quercetin 10 μM 24 h followed by 1 mM ATP 4 h), and MCC950 group (5 μM MCC950 1 h according to the manufacturer’s instructions and then 10 μg/mL LPS 24 h followed by 1 mM ATP 4 h). The protein expression of NLRP3, caspase-1, ASC, GSDMD, ZO-1, claudin-1, claudin-2, IL-1β, IL-18, and IL-6 was evaluated by western blot as explained in the related paragraph.

### Animal experiment

A total of 36 6-week-old specific pathogen free (SPF) female KM mice were purchased from Sibf (Beijing) Biotechnology Co., Ltd. and maintained in pathogen-free cages (six mice per cage), individually ventilated with negative pressure at the China Agricultural University Laboratory Animal House. Mice were housed under controlled environmental conditions (25°C and 12:12-h light-dark cycle), with water ad libitum and fed with a 6 g standard diet per day. The mice were randomly divided into six groups (*n* = 6 per group): 1) control group, which received an intraperitoneal injection of 0.9% normal saline once a day for 14 days; 2) LPS group, which also received an intraperitoneal injection of 0.9% normal saline once a day for 14 days and an intraperitoneal injection of LPS (6 mg/kg) on day 15; 3) *Cacumen Platycladi* high (CH) group, which received 1.026 mg/g aqueous extract of *Cacumen Platycladi* by gavage once a day for 14 days and an intraperitoneal injection of LPS (6 mg/kg) on day 15; 4) *Cacumen Platycladi* low (CL) group, which received 0.516 mg/g aqueous extract of *Cacumen Platycladi* by gavage once a day for 14 days and an intraperitoneal injection of LPS (6 mg/kg) on day 15; 5) quercetin high (QH) group, which received 50 mg/kg quercetin by gavage once a day for 14 days and an intraperitoneal injection of LPS (6 mg/kg) on day 15; 6) quercetin low (QL) group, which received 10 mg/kg quercetin by gavage once a day for 14 day and an intraperitoneal injection of LPS (6 mg/kg) on day 15. The protocol is shown in Fig. 5c.

The stools were collected after 12 h of fasting (only water was provided) from the treatment with LPS, to evaluate the fecal status (diarrhea) and fecal occult blood. The blood was collected at the end of the experiment on day 15 from the orbital vein, and the number of inflammatory cells such as white blood cells (WBC), neutrophils (Neu), lymphocytes (Lym), and monocytes (Mon) was measured using an automatic blood analyzer (C-5000VET, Beijing, China).

The mice were anesthetized in a sterile environment and euthanized with spinal dislocation at the end of the experiment, and their small intestine and colon were immediately collected. A fraction of the collected tissues was fixed in 4% formaldehyde and embedded in paraffin for subsequent analysis, while the other fraction was washed with ice-cold sterilized saline and stored in liquid nitrogen at −80°C for further analysis by western blot and RT-qPCR. The Disease Activity Index (DAI), which included the degree of weight loss, was also measured, according to a previous report (31).

### Histology

The small intestine and colon were fixed in 4% formalin, maintained at room temperature for 48 h, and then dehydrated with different concentrations of alcohol. The dehydrated tissues were then embedded in paraffin, and 5 μm-thick sections were cut. The sections were mounted on slides, dried in an oven at 60°C for 30 min, and stained with hematoxylin and eosin (H&E) (Beyotime, Beijing, China), according to a standard protocol. The histopathological changes in the small intestine and colon tissues were observed under a light microscope (Olympus BX41, Tokyo, Japan) at 10X, 20X, and 40X magnification.

### Statistical analysis

Statistical analysis was performed using the SPSS v19.0 software and expressed as mean ± standard error (mean ± standard error of measurement [SEM]). Different groups were compared using one-way analysis of variance (ANOVA) with Bonferroni correction as post-hoc testing. A value of *P* < 0.05 was considered statistically significant.

## Results

### Effect of quercetin on the viability and migration of RIMVECs

**Fig. 1:**
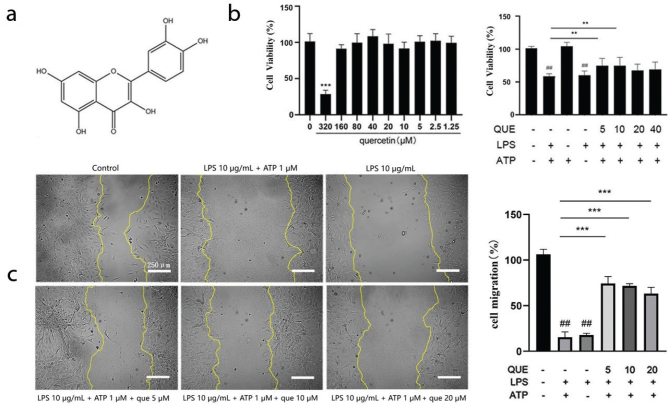
Effect of quercetin on the viability and migration of rat intestinal microvascular endothelial cells (RIMVECs). (a) Chemical structure of quercetin. Chemical formula, C15H10O7; molecular weight = 302.236. CAS number: 117-39-5. (b) Effect of quercetin on the viability of RIMVECs (*n* = 6). Results are expressed as mean ± SEM. **P < 0.01 versus LPS + ATP group, ^##^*P* < 0.01 versus control group. (c) Effect of quercetin on the migration of RIMVECs at 24 h. Scale bar: 250 μm (40 × magnification, *n* = 3). Results are expressed as mean ± SEM. ***P < 0.001 versus LPS+ATP group and ^##^*P* < 0.01 versus control group.

No significant difference was observed in the proliferation of RIMVECs treated with all the doses of quercetin after 48 h as compared to the control group except for the treatment at the dose of 320 μM, which significantly reduced the cell survival rate (*P* < 0.001); thus, this dose was not considered anymore (Fig. 1b). The viability of RIMVECs decreased after the co-stimulation with LPS and ATP as well as after LPS alone (LPS+ATP 53.91% and LPS 54.07%, *P* < 0.01 for both groups compared to the control group). However, the treatment with 5 and 10 μM quercetin effectively improved the survival rate of RIMVECs (5 μM 66.37% and 10 μM 65.15%, *P* < 0.01; Fig. 1b). In addition, the co-stimulation with LPS-ATP and LPS alone for 24 h inhibited the migration of RIMVECs (*P* < 0.01, compared with the control). The 5, 10, and 20 μM quercetin treatment increased the migration of RIMVECs (*P* < 0.001 with all doses), with a migration rate of 67, 65, and 59%, respectively (Fig. 1c).

### Effect of quercetin on the NLRP3 signaling pathway in RIMVECs

**Fig. 2:**
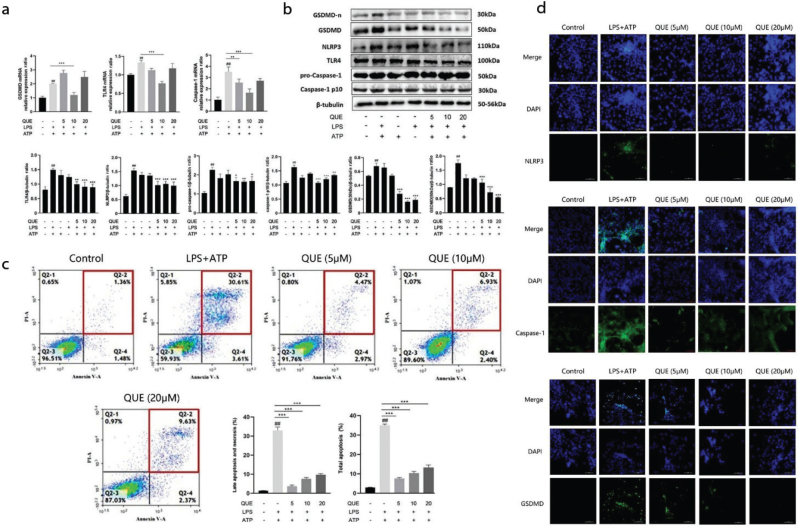
Effect of quercetin on the NOD-like receptor 3 (NLRP3) signaling pathway in RIMVECs. (a) Effect of quercetin on GSDMD, TLR4, and caspase-1 mRNA expression in RIMVECs incubated with LPS+ATP in the presence or absence of quercetin, as measured by RT-qPCR (*n* = 3). Results are expressed as mean ± SEM. ^##^*P* < 0.01 versus control group, **P < 0.01 and ***P < 0.001 versus LPS+ATP group. (b) Effect of quercetin on the expression of NLRP3 and pyroptosis-related proteins: TLR4, NLRP3, pro-caspase-1, caspase-1, and GSDMD protein expression (*n* = 3) in RIMVEC incubated with LPS+ATP in the presence or absence of quercetin, as measured by western blot. Results are expressed as mean ± SEM. ^#^*P* < 0.01 versus control group, *P < 0.05, **P < 0.01, and ***P < 0.001 versus LPS+ATP group. (c) Effect of quercetin on pyroptosis in RIMVECs incubated with LPS+ATP in the presence of quercetin measured by flow cytometry (*n* = 3). Results are expressed as mean ± SEM. ^##^*P* < 0.01 versus control group, ***P < 0.001 versus LPS+ATP group. (d) Effect of quercetin on the pyroptosis-related proteins TLR4, caspase-1, and GSDMD in RIMVEC incubated with LPS+ATP in the presence or absence of quercetin, as measured by immunofluorescence (*n* = 3).

*TLR4* and *GSDMD* mRNA expression significantly increased in the LPS-ATP group (TLR4: LPS+ATP 1.33; GSDMD: LPS+ATP 1.97, *P* < 0.01 compared to the control; Fig. 2a), but quercetin 10 μM significantly reduced their expression (TLR4: 10 μM 0.74; GSDMD: 10 μM 1.12, *P* < 0.001; Fig. 2a). In addition, quercetin 5 and 10 μM reduced the mRNA expression of Caspase-1 in RIMVECs (2.21, *P* < 0.01 and 1.54, *P* < 0.001, respectively), which was increased by the LPS-ATP treatment (3.47, *P* < 0.01) (Fig. 2a). The treatment with LPS+ATP significantly increased TLR4, NLRP3, pro-Caspase-1, Caspase-1, and GSDMD protein expression as compared to the control group (*P* < 0.01 for all proteins compared to the control). However, the treatment with quercetin at 5, 10, and 20 μM reduced their expression (*P* < 0.05, *P* < 0.01, and *P* < 0.001; Fig. 2b).

The same groups were considered to evaluate whether the expression of the above genes and proteins was associated with the occurrence of pyroptosis, and the effect of quercetin on pyroptosis was also evaluated. The percentage of late apoptotic cells and necrotic cells in the co-stimulated LPS-ATP group was increased compared to that in the control group (30.61% vs. 1.36%, *P* < 0.01). However, the 5, 10, and 20 μM quercetin treatment significantly decreased the percentage of late apoptosis and necrosis in RIMVECs (4.47, 6.93, and 9.63%, *P* < 0.001 for all doses; Fig. 2c).

The distribution and expression of NLRP3, GSDMD, and caspase-1 proteins were evaluated by immunofluorescence, which showed the high expression of these proteins in most of the cells in the LPS-ATP group. Nevertheless, the treatment with 5, 10, and 20 μM quercetin decreased their expression (Fig. 2d).

### Effect of quercetin and MCC950 on the NF-κB signaling pathway, pyroptosis-related proteins, tight junction proteins, and pro-inflammatory cytokines in RIMVECs

**Fig. 3:**
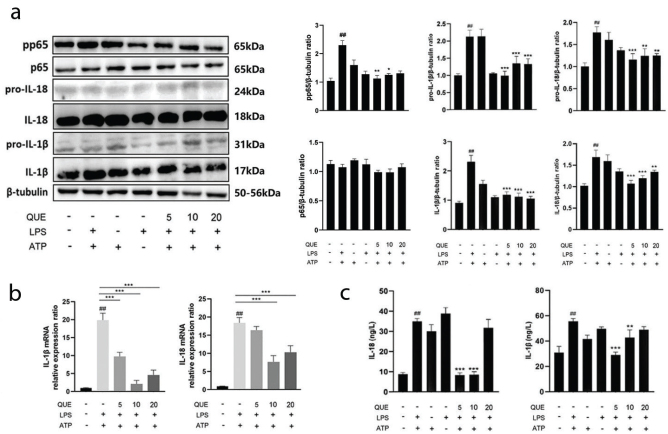
Effect of quercetin on the nuclear factor-kappa B (NF-κB) signaling pathway and pro-inflammatory cytokine expression in RIMVECs. (a) p65, p-p65, pro-IL-18, IL-18, pro-IL-1β, and IL-1β protein expression (*n* = 3) in RIMVEC incubated with LPS+ATP in the presence or absence of quercetin as measured by western blot. Results are expressed as mean ± SEM. ^##^*P* < 0.01 versus control group, *P < 0.05, **P < 0.01, and ***P < 0.001 versus LPS+ATP group. (b) IL-1β and IL-18 mRNA expression in RIMVECs incubated with LPS+ATP in the presence or absence of quercetin, as measured by RT-qPCR. Results are expressed as mean ± SEM. ^##^*P* < 0.01 versus control group, ***P < 0.001 versus LPS+ATP group (*n* = 3). (c) IL-1β and IL-18 protein levels in the medium of RIMVEC incubated with LPS+ATP in the presence or absence of quercetin, as measured by ELISA (*n* = 3). Results are expressed as mean ± SEM. ^##^*P* < 0.01 versus control group, **P < 0.01 and ***P < 0.001 versus LPS+ATP group.

The NF-κB is an important inflammatory signaling pathway, which activates the NLRP3 inflammasome. The expression of p-p65 in the co-stimulated LPS+ATP group significantly increased compared to the expression in the control (2.14, *P* < 0.01). However, the treatment with 5 and 10 μM quercetin reduced the expression of p-p65 (1.04 and 1.25, respectively, *P* < 0.05 and *P* < 0.01) (Fig. 3a).

**Fig. 4:**
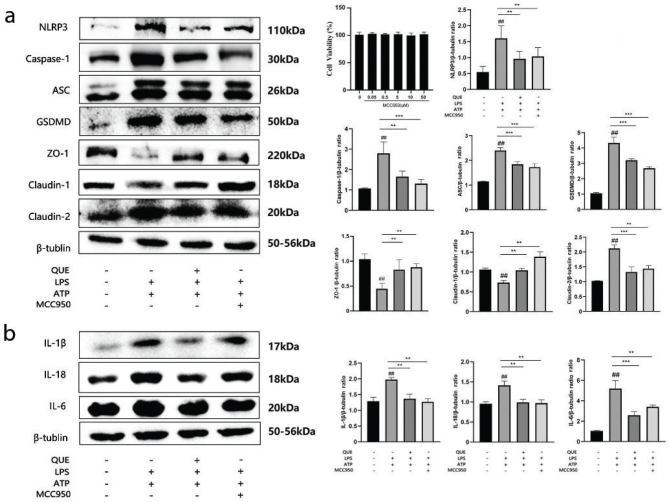
Effect of quercetin and MCC950 on the expression of pyroptosis-related proteins, tight junction proteins, and inflammatory cytokines in RIMVECs (*n* = 3). (a) Changes in the expression of pyroptosis-related proteins (NLRP3, caspase-1, ASC, and GSDMD) and tight junction proteins (ZO-1, claudin-1, and claudin-2) (*n* = 3) in RIMVEC incubated with LPS+ATP in the presence or absence of quercetin, as measured by western blot. Results are expressed as mean ± SEM. ^##^*P* < 0.01 versus control group, **P < 0.01 and ***P < 0.001 versus LPS+ATP group. (b) IL-18, IL-1β, and IL-6 protein expression in RIMVECs incubated with LPS+ATP in the presence or absence of quercetin, as measured by western blot. Results are expressed as mean ± SEM. ^##^*P* < 0.01 versus control group, **P < 0.01 and ***P < 0.001 versus LPS+ATP group.

IL-1β and IL-18 gene and protein expression were also assessed to evaluate the consequences on the effect of NF-kB explained above. The LPS+ATP group showed an increase in the protein expression of IL-1β and IL-18 in RIMVECs, as well as an increase of the precursor (*P* < 0.01 compared to the control), but their increase was inhibited by quercetin at all doses (*P* < 0.01 and *P* < 0.001) (Fig. 3a). The same result was obtained by the analysis of IL-1β and IL-18 mRNA expression (*P* < 0.001 for all doses of quercetin) (Fig. 3b). The results on the secretion of the protein in the medium of RIMVECs evaluated by ELISA confirmed the results by western blot, except for the dose of 20 μM quercetin that did not reduce the level of IL-1β and IL-18 in the medium (Fig. 3c). The use of MCC950, an inhibitor of NLRP3, showed results similar to those obtained by quercetin, since the protein expression of pyroptosis-related proteins, tight junction proteins (*P* < 0.01 and *P* < 0.001 compared with the LPS+ATP group; Fig. 4a), and inflammatory factors (*P* < 0.01 compared with the LPS+ATP group; Fig. 4b) were significantly reduced by MCC950. MCC950 did not exert any effect on the viability of RIMVECs (Fig. 4a).

### Mouse DAI values, blood results, and histopathology of the intestine

**Fig. 5:**
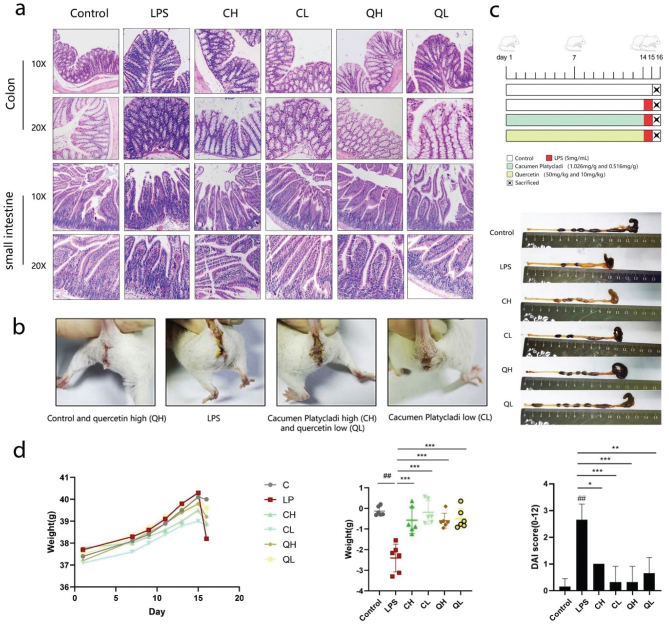
Histopathology of the intestine, fecal status, and body weight of the mice treated with LPS, quercetin, and *Cacumen Platycladi*. (a) Histopathology of the small intestine and colon (*n* = 6). (b) Changes of fecal status in the different groups. (c) Experimental protocol and changes in the colon and cecum length. (d) Mouse body weight. Results are expressed as mean ± SEM. ^##^*P* < 0.01 versus control group, *P < 0.05, **P < 0.01, and ***P < 0.001 versus LPS group.

The severity of enteritis was determined by calculating the DAI, which included the degree of weight loss (*P* < 0.001; Table 2 and Fig. 5d), fecal status (Fig. 5b), and fecal occult blood. The results revealed that LPS reduced the colon and cecum length, and caused the presence of more fecal occult blood, severe diarrhea, and enteritis as compared to the *Cacumen Platycladi* groups (Quercetin can be used as an index for the determination of carbon content in *Cacumen Platycladi*) and Quercetin groups (Fig. 5c), which reduced these symptoms. These results revealed that the body weight significantly changed, and the DAI score indicated that LPS increased it, while quercetin and *Cacumen Platycladi* significantly reduced the DAI score.

**Table 2:** Mouse Disease Activity Index (DAI).

Group	*n*	D0	D7	D14	D15	Weight loss score
Control	6	37.4 ± 0.78	38.1 ± 1.12	40.1 ± 0.93	40.0 ± 0.88	0.25% (0)
LPS	6	37.7 ± 0.82	38.3 ± 0.85	40.3 ± 1.02	38.2 ± 0.91	5.21% (2)##
CH	6	37.8 ± 0.66	38.0 ± 0.76	39.5 ± 0.68	38.9 ± 0.75	1.52% (1)***
CL	6	37.1 ± 1.14	37.6 ± 1.07	39.0 ± 0.81	38.8 ± 0.69	0.51% (0)***
QH	6	37.2 ± 0.80	38.1 ± 0.77	39.8 ± 0.97	39.2 ± 1.07	1.51% (0)***
QL	6	37.5 ± 1.07	38.2 ± 1.00	40.1 ± 0.94	39.6 ± 0.85	1.25% (1)***

Results are expressed as mean ± SEM.

## *P* < 0.01 versus control group.

*** *P* < 0.001 versus LPS group.

Since LPS caused severe colon injury, the morphology of the colon tissue was evaluated, revealing an incomplete structure in the colon of the LPS group mice, which was not observed in those of *Cacumen Platycladi* and quercetin group mice (Fig. 5a). In addition, the lymph nodes on the surface of the intestinal mucosa of the LPS group mice were enlarged, accompanied by the destruction of the mucosal structure and infiltration of a large number of inflammatory cells. However, the colon tissue of the *Cacumen Platycladi* and quercetin groups showed mild symptoms.

The number of WBC, Neu, Lym, and Mon were significantly higher in the LPS group as compared to the other groups (*P* < 0.01; Table 3). Among these groups, CL and QH showed a better protective effect, although all the doses were protective.

**Table 3:** Mouse blood parameters.

	WBC (×10^9^/L)	Neu (×10^9^/L)	Lym (×10^9^/L)	Mon (×10^9^/L)
Control	5.07 ± 0.22	0.40 ± 0.03	5.98 ± 0.07	0.10 ± 0.01
LPS	13.20 ± 1.19 ##	1.83 ± 0.54 **##**	14.46 ± 0.10 **##**	0.32 ± 0.02 **##**
CH	9.08 ± 0.46**	0.75 ± 0.08***	9.92 ± 0.06***	0.21 ± 0.01**
CL	5.48 ± 0.31***	0.60 ± 0.21***	6.54 ± 0.02***	0.18 ± 0.01***
QH	5.52 ± 0.75***	0.48 ± 0.17***	6.18 ± 0.14***	0.12 ± 0.02***
QL	7.04 ± 0.44***	0.51 ± 0.49***	7.98 ± 0.05***	0.20 ± 0.01**

Results are expressed as mean ± SEM.

## *P* < 0.01 versus control,

** *P* < 0.01 and

*** *P <* 0.001 versus LPS group.

### Effect of *Cacumen Platycladi* and quercetin on the NLRP3 signaling pathway in the mouse gut

**Fig. 6:**
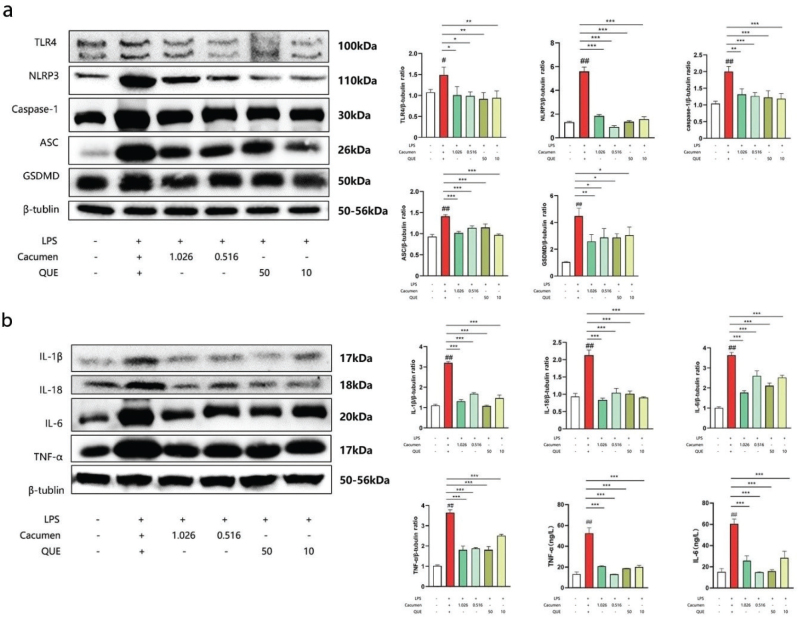
Effect of *Cacumen Platycladi* and quercetin on the NLRP3 signaling pathway in the mouse gut. (a) TLR4, NLRP3, caspase-1, ASC, and GSDMD protein expression in the gut measured by western blot. (b) IL-18, IL-1β, IL-6, and TNF-α protein expressions measured by western blot; TNF-α and IL-6 protein expressions in the gut measured by ELISA. Results are expressed as mean ± SEM. *P < 0.05, **P < 0.01, and ***P < 0.001 versus LPS group. ^##^*P* < 0.01 versus control group.

TLR4, NLRP3, caspase-1, ASC, and GSDMD protein expressions were assessed to confirm the effectiveness of *Cacumen Platycladi* and Quercetin in the inhibition of pyroptosis in the mouse gut. Their protein expression significantly increased in the LPS group (1.48, 5.86, 1.99, 1.45, and 4.23, *P* < 0.05 and *P* < 0.01) as compared to their expression in the control group (1.06, 1.22, 1, 0.93, and 1). However, *Cacumen Platycladi* and quercetin significantly reduced the expression of TLR4 (*P* < 0.05 and *P* < 0.01, respectively), NLRP3 (*P* < 0.001 for both compounds at all doses), caspase-1 (*P* < 0.01 and *P* < 0.001 for the two doses of *Cacumen Platycladi* and *P* < 0.001 for quercetin), ASC (*P* < 0.001 for both compounds at all doses), and GSDMD (*P* < 0.05 and *P* < 0.01 for the two doses of *Cacumen Platycladi* and *P* < 0.05 for quercetin) as compared to their expression in the LPS group (Fig. 6a).

The effect of *Cacumen Platycladi* and Quercetin treatment on inflammation was also evaluated by measuring the expression of inflammation-related proteins. IL-18, IL-1β, IL-6, and TNF-α protein expressions in the LPS group were significantly increased as compared to the control group (*P* < 0.01; Fig. 6b). However, both *Cacumen Platycladi* and quercetin significantly reduced the expression of IL-18, IL-1β, IL-6, and TNF-α as compared to their expression in the LPS group (*P* < 0.001). TNF-α and IL-6 protein expressions measured by ELISA also showed similar results as those obtained by western blot (*P* < 0.001) (Fig. 6b).

## Discussion

**Fig. 7:**
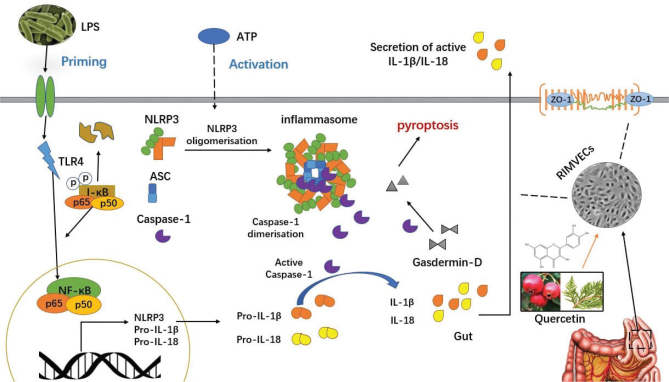
*Cacumen Platycladi* and quercetin inhibit the NLRP3 signaling pathway in the gut. A model of the protective effect of *Cacumen Platycladi* and quercetin in LPS-induced cell inflammation and pyroptosis was hypothesized. LPS results in inflammation and pyroptosis in the intestine of mice. Quercetin found in food and traditional Chinese medicine reduces caspase-1, ASC, and GSDMD by downregulating the TLR4/NF-κB/NLRP3 pathway, and reduces the recruitment of IL-18, IL-1β, and other inflammatory factors. In addition, it upregulates the tight junction protein ZO-1 to enhance the barrier function of the intestinal tract. Thus, quercetin protects against LPS-induced cell pyroptosis and death through this mechanism.

LPS plays an important role in mediating intestinal inflammation. The inflammatory damage induced by LPS alone on RIMVECs needs further stimulation by ATP to activate inflammasome and pyroptosis; therefore, the co-stimulation of LPS and ATP was used to induce RIMVECs for the establishment of a pyroptosis model (32). Quercetin is an active ingredient of *Cacumen Platycladi* with several protective effects including bacterial infections (33, 34). In this study, quercetin significantly reduced pyroptosis, the expression of inflammatory factors, and the destruction of the intestinal barrier caused by LPS by downregulating the activation of the NLRP3 inflammasome in RIMVECs (Fig. 7). The animal experiments further confirmed these results, revealing that quercetin protected intestinal cells from pyroptotic inflammation, thus maintaining gut health.

The intestinal infection by bacteria alters the structural integrity of MVECs, causing the production of many inflammatory factors. LPS infection induces pyroptosis, thus disturbing the barrier function of endothelial cells and increasing the permeability of the cell membrane. Quercetin significantly suppressed pyroptosis, as suggested by the inhibition of NLRP3, caspase-1, and GSDMD. Tight junction is closely related to cell growth and migration. Its inhibition or disappearance causes a delay in cell migration and proliferation, consequently inhibiting wound healing (17, 35). ZO-1 is widely used to determine the damage of the intestinal mucosal barrier (36, 37). The present study showed that the co-stimulation of LPS-ATP decreased the viability and migration of RIMVECs, consequently inhibiting the heal of the physical scratches. The co-stimulated LPS-ATP group showed a reduction in ZO-1 expression, while quercetin prevented its reduction. These results suggested that ZO-1, which is originally distributed around the cell membrane, was destroyed, thus compromising the junction between cells of the intestinal barrier, while quercetin restored the expression of this protein, thus restoring the structure of the intestinal barrier and effectively enhancing the ability of cell migration, which, from another perspective, could be considered as a restored ability to heal wounds. These results were consistent with those obtained after inhibiting the expression of NLRP3 with the inhibitor; it also increased the protein expression of claudin-1 and decreased that of claudin-2 in cells. Indeed, claudin-1 is involved in TJ integrity, suggesting that its expression is fundamental for a normal intestinal barrier structure and correct function (38). Claudin-2 is unregulated in inflammatory bowel disease (IBD), and its deficiency reduces colitis progression and barrier defect, suggesting that its reduced expression prevents the inflammation of the intestinal barrier (39).

Apoptosis and necrosis in intestinal diseases are related to the massive infiltration of inflammatory cells and release of pro-inflammatory factors, triggering to pyroptosis (40). Our results showed that quercetin significantly reduced the number of late apoptotic and necrotic cells and blocked the pyroptosis signaling to protect RIMVECs from infection. Quercetin reduces pyroptosis and consequent inflammation and oxidative stress in several diseases (41, 42), but few studies evaluated the mechanism of pyroptosis in enteritis, which is shown in this work, at least in part. However, since no references are available on the doses of quercetin in the treatment of enteritis, one should keep in mind that excessive quercetin might cause abnormal estrogen levels and genetic toxicity in the body (43).

NF-κB activates NLRP3 and upregulates its expression (44, 45), consequently promoting pyroptosis (46). TLR4 on the surface of endothelial cells recognizes and binds to LPS and activates the NLRP3 inflammasome through the TLR4/NF-κB signaling pathway. TLR4 further activates caspase-1 to cleave GSDMD, causing pyroptosis-related cell rupture and pore formation in the cell membrane (12, 47, 48), as well as the release of IL-1β and IL-18 that enhance inflammation. The activation of NF-κB also inhibits the transcription of tight junction proteins, thereby affecting the normal structure, morphology, growth, and migration of cells (49–51). Our results are consistent with these reports, since the co-stimulation of LPS and ATP promoted the activation of NF-κB p65 and increased the expression of IL-1β, IL-18, and IL-6. Nevertheless, quercetin decreased the expression of NF-κB p65 and significantly downregulated the protein expression, secretion, and mRNA of IL-1β, IL-18, and IL-6.

Since our experiments *in vitro* revealed the anti-inflammatory and immunomodulatory properties of quercetin (52), *Cacumen Platycladi*, which contains quercetin, was selected for the *in vivo* experiment due to its use in the traditional Chinese medicine. Its composition is complex; thus, its effect has similarities and differences to that of quercetin monomer. Therefore, both *Cacumen Platycladi* and quercetin were used in our *in vivo* experiments, to evaluate the similarities and/or differences between the two, since only few studies are available on the effects of *Cacumen Platycladi* in human health (53, 54), despite being a long-standing medicine.

TLR4, NLRP3, caspase-1, GSDMD, and ASC protein expression increased after the injection of LPS, indicating the activation of NLRP3 signaling pathway in the colon. Nevertheless, their expression significantly decreased in the quercetin and *Cacumen Platycladi* groups, showing their anti-pyroptotic effect. Moreover, quercetin inhibited IL-1β, IL-18, IL-6, and TNF-α protein expression as well as the secretion of IL-6 and TNF-α, consequently reducing inflammation.

The effects of quercetin on the intestinal immunity of the mice were further analyzed by the blood routine tests, which showed that the number of WBC, Neu, Lym, and Mon significantly increased in the LPS group as compared to those in the control group. These immune cells are involved in the immune response of acute LPS infection, suggesting the presence of inflammation and bacterial infection (55). From the pathological point of view, edema, thinner intestinal wall, shorter total length of the intestine, excess in the infiltration of inflammatory cells, and destroyed structure of small intestinal villi were observed in the colon and small intestine of the LPS-treated mice. However, the mice treated with *Cacumen Platycladi* and quercetin showed a better intestinal structure, less diarrhea, and a lower rate of fecal occult blood. In general, both the aqueous extract of *Cacumen Platycladi* and quercetin significantly improved pyroptosis and inflammation in the intestines of mice, although the correlation between the concentration and DAI score of *Cacumen Platycladi* groups was different from those of the Quercetin groups. This might be due to the poor palatability of the water extract of the traditional Chinese medicine, which needs to be improved.

In conclusion, our results revealed the potential use of this Chinese herbal medicine rich in quercetin in the treatment of bacterial enteritis and pyroptosis-related diseases. Further *in vivo* animal experiments to confirm these results could pave the way for the design of trials in human subjects suffering from bacterial enteritis using these natural substances, which are less aggressive than a standard drug treatment.
